# The Very Efficient Assessment of Need for Cognition: Developing a Six-Item Version*

**DOI:** 10.1177/1073191118793208

**Published:** 2018-08-10

**Authors:** Gabriel Lins de Holanda Coelho, Paul H. P. Hanel, Lukas J. Wolf

**Affiliations:** 1 Cardiff University, Cardiff, UK; 2 University of Bath, Bath, UK

**Keywords:** need for cognition, short scale, psychometrics, validation, item response theory

## Abstract

The need for cognition refers to people’s tendency to engage in and enjoy thinking and has become influential across social and medical sciences. Using three samples from the United States and the United Kingdom (*N* = 1,596), we introduce a six-item short version of the Need for Cognition Scale (NCS-18). First, we reduced the number of items from 18 to 6 based on the items’ discrimination values, threshold levels, measurement precision (item information curve), item–total correlations, and factor loadings. Second, we confirmed the one-factor structure and established measurement invariance across countries and gender. Finally, we demonstrated that while the NCS-6 provides significant time savings, it comes at a minimal cost in terms of its construct validity with external variables such as openness, cognitive reflection test, and need for affect. Overall, our findings indicate that the NCS-6 is a parsimonious, reliable, and valid measure of need for cognition.

The need for cognition is a stable personality trait that describes individuals’ tendency to engage in and enjoy effortful cognitive activity (Cacioppo & Petty, 1982). Individuals high in the need for cognition tend to seek out and reflect on information to make sense of stimuli and events, whereas individuals low in the need for cognition tend to use other sources such as heuristics to make sense of the world. Thus, given this tendency to seek out and enjoy effortful cognitive activity, those higher in need for cognition are generally expected to have more positive attitudes toward situations that require reasoning and problem solving, and to respond more substantively to such situations (Cacioppo, Petty, Feinstein, & Jarvis, 1996).

Since the 1950s, the need for cognition has attracted considerable research attention (Cacioppo et al., 1996; Cohen, Stotland, & Wolfe, 1955) and has been studied in various areas within psychology, including clinical (Bagby, Taylor, & Ryan, 1986), social (Aquino, Haddock, Maio, Wolf, & Alparone, 2016; Wolf, von Hecker, & Maio, 2017), personality (Sadowski & Cogburn, 1997), and educational psychology (Kardash & Scholes, 1996), and in areas beyond psychology, such as management (Kearney, Gebert, & Voelpel, 2009), journalism (Liu & Eveland, 2005), and marketing (Haddock, Maio, Arnold, & Huskinson, 2008; Haugtvedt, Petty, & Cacioppo, 1992). This decades-long research has consistently shown that the need for cognition is meaningfully related to a wide range of individual difference variables, and to attitudinal and behavioral outcomes (e.g., Cacioppo et al., 1996).

The assessment of the need for cognition has so far generally required the original 34-item version (Cacioppo & Petty, 1982) or a shortened 18-item version (Cacioppo, Petty, & Kao, 1984). However, researchers have long recognized the dearth of and need for an even shorter measure of need for cognition, as evidenced by several studies that have simply selected the highest loading items from the longer versions (e.g., Bullock, 2011; Davis, Severy, Kraus, & Whitaker, 1993), or studies using an unvalidated two-item measure (Bakker & Lelkes, 2016; Bizer, Krosnick, Petty, Rucker, & Wheeler, 2000). To fill this gap, the present research developed and validated a very short scale to measure the need for cognition, the NCS-6, to further enhance its practicality. We tested the usefulness of the NCS-6 across two countries, the United States and the United Kingdom.

## Need for Cognition Scale

The Need for Cognition Scale (NCS) was developed by Cacioppo and Petty (1982), drawing on earlier work by Cohen and others (e.g., Cohen et al., 1955). To develop the scale, Cacioppo and Petty generated 45 items and subsequently selected those 34 items that discriminated between an a priori chosen high need for cognition group (university faculty members) and a low need for cognition group (assembly line workers). By reducing the number of items, Cacioppo et al. (1984) developed a more practical 18-item version of the NCS which correlated very highly with the NCS-34 (*r* = .95). Since its development, the two articles that introduced the long and short version of need for cognition (Cacioppo & Petty, 1982; Cacioppo et al., 1984) have together been cited more than 8,600 times (Google Scholar, April 2017), attesting to the measure’s importance and popularity in scientific research. Researchers have since then validated the NCS-34 and NCS-18 in various languages and/or countries, including Australia (Forsterlee & Ho, 1999), Germany (Bless, Wänke, Bohner, Fellhauer, & Schwarz, 1994), Greece (Georgiou & Kyza, 2019), Spain (Gutiérrez-Maldonado, Bajén, Sintas, & Amat, 1993), Taiwan (Kao, 1994), Portugal (Silva & Garcia-Marques, 2013), Netherlands (Pieters, Verplanken, & Modde, 1987), Brazil (Gouveia, Mendes, Soares, Monteiro, & Santos, 2015), and in a U.S.-Hispanic sample (Culhane, Morera, & Hosch, 2004), and it has been adapted to different populations, including children and adolescents (Keller et al., 2019). The NCS consistently exhibited high internal consistencies, with reliabilities generally varying between α = .80 and α = .90 (Cacioppo et al., 1996), and was found to be invariant across age groups (Soubelet & Salthouse, 2016).

There is abundant research supporting the scientific importance of the NCS, showing its meaningful relations to other individual difference variables. For instance, the need for cognition is positively linked with openness to experience and intelligence (Furnham & Thorne, 2013), desire for control (Thompson, Chaiken, & Hazlewood, 1993), intrinsic motivation (Cacioppo et al., 1996), information processing (Sicilia, Ruiz, & Munuera, 2005), and many other variables (Cacioppo et al., 1996). In contrast, it is negatively related to neuroticism, external locus of control, and dogmatism, and unrelated to loneliness, shyness, and sociability (see, Cacioppo et al., 1996, for an overview). Importantly, the need for cognition also predicts a range of attitudinal and behavioral outcomes, including preferences for a complex number-circling task over a simple one (Cacioppo & Petty, 1982), achieving higher grade point averages (see, Cacioppo et al., 1996), interpersonal and intergroup attitudes (Aquino et al., 2016; Wolf et al., 2017), persuasion (Haddock et al., 2008), news media skepticism (Tsfati & Cappella, 2005), and responses toward sexual and nonsexual appeals (Putrevu, 2008).

## Why Shorter Measures?

There are recent calls to develop shorter scales to assess psychological constructs (e.g., Coelho et al., 2018; Gosling, Rentfrow, & Swann, 2003; Rammstedt & Beierlein, 2014). Longer scales can be problematic in several ways, by increasing participant fatigue, lack of attention, boredom, and dropout rates, which in turn may influence the quality of the data (e.g., lower reliability and validity levels, missing data; Rammstedt & Beierlein, 2014), and may bias participants’ cognitive and emotional processes (Tourangeau, Rips, & Rasinski, 2000). In other words, researchers often prefer shorter scales. For example, the short version of the Need for Affect Scale (NFA; Appel, Gnambs, & Maio, 2012) reduced the original 26-item scale (Maio & Esses, 2001) to 10 items and has been used frequently since then (>10 citations/year). These and many other examples (e.g., Back et al., 2013; Lo, Zhao, Kwok, Chan, & Chan, 2017), show that researchers often prefer using shorter versions of the original scales, even if the absolute reduction is only 10 to 20 items.

Given the popularity and theoretical importance of the NCS, it is useful to reduce the number of items to enhance the NCS’s practicality in scientific research. As the measure contains items that require more attention from the participants because of their length and complexity (e.g., Item 15: *I would prefer a task that is intellectual, difficult, and important to one that is somewhat important but does not require much thought*, Item 5: *I try to anticipate and avoid situations where there is a likely chance I will have to think in depth about something*), its use may likely result in participant fatigue, lack of attention, boredom, and dropout, especially if the NCS is part of a larger battery of tests.

As indicated above, researchers often relied on shorter, yet not formally validated versions of the NCS. For instance, a two-item version was proposed for the National Election Survey pilot study (Bizer et al., 2000), asking participants to report how much they liked or disliked two newly formed need for cognition items. However, in a yet unpublished study, Bakker and Lelkes (2016) report that the NCS-2 fails to moderate the impact of policy cues on attitudes, whereas longer versions of the NCS reliably detect this moderation. The NCS-2 may also be problematic because its items are wordier than the items of the NCS-18. Similarly, in a project that was set up to replicate several classical studies (Ebersole et al., 2016), the researchers used a six-item version of the NCS. The authors did not reproduce a classic effect of need for cognition on the impact of argument strength on persuasion (Cacioppo, Perry, & Morris, 1983). Luttrell, Petty, and Xu (2017) noted methodological issues in this study, including a significantly lower reliability compared with the original NCS-18, and they demonstrated that the classic effect could be obtained with the NCS-18. A range of other studies have used short versions by selecting the highest loading items from the NCS-18 (e.g., Bullock, 2011; Davis et al., 1993; see, Cacioppo et al., 1996, for an overview). However, to the best of our knowledge, there have been no formal validations of these shorter scales.

## Current Research

In the present research, we used various statistical approaches to determine the number of items of a very short scale, going beyond previous research developing shorter versions of the NCS. For example, to create the NCS-18, Cacioppo et al. (1984) relied on the factor loadings of the original 34-item version. The authors selected the items based on their factor loadings of the NCS-34 and tested how their inclusion would affect the overall reliability of the scale. Even though this method can result in a measure with satisfactory good internal consistency, it is important to also examine other criteria. Using a more comprehensive approach that combines classical test theory (CTT) and item response theory (IRT), we considered a range of item parameters that are crucial for shortening a scale while minimizing potential costs to the psychometric properties. While the main focus of CTT is to test the reliability and validity of a measure in factor analyses of the items, IRT aims to explain the relations between item responses and the underlying construct (Cappelleri, Jason Lundy, & Hays, 2014). Hence, while CTT is necessary to demonstrate the usefulness of a scale in terms of its reliability and validity, an IRT approach complements CTT by providing more specific information about the items (e.g., discrimination, difficulty, information; Pasquali & Primi, 2003). Therefore, we used a comprehensive approach, allowing us to draw on more extensive information to form a short scale of high quality.

Similar to previous research (e.g., Rauthmann, 2013), we had no a priori expectation for the eventual number of items of the shortened scale. Instead, our approach was guided by previous recommendations for short scale developments to report the amount of participation time the scale can save compared with the original scale, in addition to ensuring that the short scale’s reliability and validity are comparable to the original scale (Rammstedt & Beierlein, 2014; Smith, McCarthy, & Anderson, 2000). Hence, we aimed to strongly reduce the number of items to save participation time, while retaining as much information as possible of the original scale to develop a useful very short NCS with strong psychometric properties.

An additional aim of this research was to examine whether our newly formed scale was invariant across participant gender and across country. The NCS-18 has previously been found to be gender-neutral and to show similar factorial structures in both Europe and North America (Cacioppo et al., 1996). However, although the one-dimensional factor structure has generally been reproduced, a few studies have found two or three factors (Forsterlee & Ho, 1999); Tanaka, Panter, & Winborne, 1988). Thus, to demonstrate that our shortened NCS allows for meaningful comparisons across these groups (Davidov, Meuleman, Cieciuch, Schmidt, & Billiet, 2014), we tested whether our shortened scale is answered in the same way by men and women, and by U.S. and U.K. participants. This test of invariance hence provides further evidence of the usefulness of our scale across contexts and participant groups.

Hence, in Study 1, we used two large samples from the United States and the United Kingdom to identify the most psychometrically sound items and reduce the number of NCS items accordingly. In addition, Study 1 examined whether our newly formed scale was invariant across participant gender and across countries. Study 2 used an independent U.K. sample to corroborate our findings using the newly developed very short NCS. Both studies tested the convergent and discriminant validity of the new NCS by examining its relations with several other variables (e.g., openness, cognitive reflection test, need for affect).

## Study 1

### Method

#### Participants

We used an American sample and a British sample. Participants in the American sample were 821 individuals (451 men; *M*_age_ = 32.12 years, standard deviation [*SD*] = 11.68), who were recruited online on Amazon’s MTurk. Participants in the British sample were 476 individuals (255 men; *M*_age_ = 38.91 years, *SD* = 12.37) who were recruited online on Prolific academic. Both samples were from the general population, and the studies were previously approved by the ethics committee.

#### Material

In addition to the NCS, both samples completed a range of convergent and divergent constructs to examine the construct validity of the NCS-6. All participants in the British sample saw the same set of questionnaires, in the order described below. The American sample consisted of subsamples who completed different measures.

##### Need for Cognition Scale (Cacioppo & Petty, 1982)

In all samples and subsamples, we administered the 18-item version (see the appendix for example items). Responses were given on a 5-point scale (1 = *extremely uncharacteristic of me*; 5 = *extremely characteristic of me*).

#### American Sample Materials

##### Need for Affect Questionnaire–short version (Appel et al., 2012)

This measure is composed of 10 items, assessing individual differences in the tendency to approach or avoid emotion-inducing situations and activities. Participants indicate to what extent they agree (−3 = *strongly disagree*; 3 = *strongly agree*) with items such as “I feel that I need to experience strong emotions regularly” (approach), and “Emotions are dangerous—they tend to get me into situations that I would rather avoid” (avoidance). The NFA scale (α = .85) and its components approach (α = .83) and avoidance (α = .87) were internally consistent. Following previous research (Appel et al., 2012; Maio & Esses, 2001), we expected a small but positive association between need for cognition and need for affect.

##### Marlowe–Crowne Social Desirability Scale (Reynolds, 1982)

This measure assesses individuals’ tendency to answer in a socially desirable way. Participants indicated for each of the 13 items (e.g., “No matter who I’m talking to, I’m always a good listener”) whether they considered it true or false. The internal consistency of this scale was good (α = .79). Based on previous research (Cacioppo et al., 1996), we expected a small positive correlation between need for cognition and social desirability.

##### Attitudes

We measured participants’ attitudes toward various social groups using a 101-point evaluation thermometer (0° = *extremely unfavorable* to 100° *extremely favorable*; Haddock, Zanna, & Esses, 1993). As described further in previous research (Wolf et al., 2017), we aggregated these scores across stereotypically warm and incompetent groups (i.e., the elderly, housewives, South Americans, children, Italian people, South American people, Irish people) and across stereotypically cold and competent groups (i.e., German people, rich people, Asian people, Jewish people, professionals, feminists). We expected that need for cognition would relate positively to attitudes toward stereotypically cold and competent groups and that it would be unrelated to attitudes toward stereotypically warm and incompetent groups (Wolf et al., 2017).

##### Attributes

We presented 24 attributes pertaining to warmth and competence (Wolf et al., 2017). Participants were asked to imagine for each attribute that they were meeting people who possessed one of these attributes. Subsequently, they were asked to evaluate these attributes on a 7-point scale (1 = *very negative*; 7 = *very positive*). We aggregated scores for all warm, cold, competent, and incompetent attributes and subsequently subtracted incompetent traits from competent traits and cold traits from warm traits to arrive at warmth and competence scores (αs > .86). We expected that need for cognition would relate positively to liking competence and that it would be unrelated to liking warmth (Wolf et al., 2017).

#### British Sample Materials

The British sample first completed the NFA scale (Approach, α = .80; Avoidance, α = .81; Overall, α = .82), followed by the Portrait Values Questionnaire (PVQ, Schwartz et al., 2001). The PVQ was developed to measure the 10 value types (e.g., conformity, self-direction) from Schwartz’s (1992) value theory. This scale consists of 21 short verbal portraits of individuals, such as “It is important to her to be rich. She wants to have a lot of money and expensive things,” which are answered on a 6-point scale (1 = *not like me at all*; 6 = *very much like me*). The internal consistencies (α) varied between .43 (for self-direction) and .77 (stimulation; median α = .66), except for tradition, where the internal consistency was very low (α = .18). Tradition was therefore not further analyzed.

We included values because of their universal importance across all social sciences and beyond (Maio, 2016). Values are usually defined as abstract ideals or principles that guide people’s behavior and transcend specific situations (Schwartz, 1992). Although the relations between need for cognition and values has not yet been researched to the best of our knowledge, we included values here because their abstract nature and transcendence of situations show conceptual overlap with need for cognition. Specifically, we expected need for cognition to be positively related to openness values (i.e., self-direction, stimulation), because the defining motivation of these values is to pursue change, and independent thoughts and actions (Schwartz, 1992). In contrast, we expected need for cognition to be negatively related to conservation values (i.e., conformity, security), because these values promote following norms and the preservation of the status quo. We had no a priori expectations about the relations between the need for cognition and the remaining values.

#### Data Analysis

All data were analyzed in SPSS and R (R Development Core Team, 2015). In SPSS, we computed descriptive statistics, the item–total correlations using Pearson’s *r*, and conducted an exploratory factor analysis (EFA) using principal axis factoring. In R, we analyzed the psychometric properties of discrimination, thresholds, and informative curves for both the individual items and the full measure, using the multidimensional item response theory (MIRT) package (Chalmers, 2012). Within the MIRT analysis, we used the graded response model, because of the polytomous nature (more than two answer categories) of the measure (Samejima, 1968). This model is well established in IRT, because it allows using all the information from the items which in turn results in a psychometrically adequate measure (Jiang, Wang, & Weiss, 2016).

Subsequently, we conducted a confirmatory factor analysis (CFA), using the *lavaan* package (Rosseel, 2012) and the robust maximum likelihood (MLR) estimator. This estimator is known for its robustness against nonnormality of data, as well as for its fit to categorical–ordinal data when the scale has five or more points (Rhemtulla, Brosseau-Liard, & Savalei, 2012). The following indices were considered (Hair, Black, Babin, & Anderson, 2015; Hooper, Coughlan, & Mullen, 2008; Tabachnick & Fidell, 2013): (1) chi-square (χ^2^), which should be nonsignificant but is sensitive to sample size; (2) comparative fit index (CFI); (3) Tucker–Lewis index (TLI), each of which need to be higher than .90 for a good model fit; (4) root mean square error of approximation (RMSEA), which should be less than .08; (5) Akaike information criterion (AIC); and (6) Bayesian information criterion (BIC), for which smaller numerical values indicate better fit.^1^

It is important to highlight that we performed the EFA on the same data sets as the CFA in Study 1. Although the unidimensional structure of the NCS has been widely reproduced, some studies found a two- or three-factor structure (Forsterlee & Ho, 1999; Tanaka et al., 1988). Thus, to ensure that the unidimensionality holds in our samples, and also to obtain the factor loadings, we conducted the EFA. We conducted the CFA to be able to compare the fit with the data for the NCS-18 and the reduced scale.

To reduce the number of items, we considered a range of criteria that are commonly used in the literature (e.g., Coelho et al., 2018; Edelen & Reeve, 2007; Peters, Sunderland, Andrews, Rapee, & Mattick, 2012; Rauthmann, 2013; Vilar, Milfont, Araújo, Coelho, & Gouveia, 2018). It is recommended that items should neither be too easy nor too difficult, have high item–total correlations, high discrimination, not be redundant with other items, and substantially contribute to the scale (informativeness). These criteria ensure that only the most reliable items are included in the final short scale. In addition, it is important to confirm that the resulting shortened scale is of sufficiently high quality by testing whether its reliability and construct validity are high and comparable to the full scale.

Finally, we performed a multigroup CFA to assess measurement invariance for gender and country. The test for measurement invariance allows us to assess how consistent participants from different groups respond to the measure. Achieving measurement invariance is necessary to allow meaningful comparisons between the chosen groups (Davidov et al., 2014), and to not end up comparing “chopsticks with forks” (F. F. Chen, 2008). This can provide benefits, for instance, in cross-cultural research regarding need for cognition. To test for invariance, we considered three models (Milfont & Fischer, 2010): (1) configural invariance, which requires the factorial structure to be invariant across groups; (2) metric invariance, which requires the loadings between observed items and latent variables to be invariant across groups; and (3) scalar invariance, which requires the indicator intercepts to be invariant across groups. The following parameters were used as thresholds: ΔCFI and ΔRMSEA, which must be equal to or less than .010 and .015, respectively (F. F. Chen, 2007), when a model is compared with the next higher one (e.g., comparing a model assuming configural invariance with a model assuming metric invariance).

### Results

#### Psychometric Properties of the NCS-18

##### Descriptive statistics and item–total correlation

Table 1 presents the descriptive statistics for all items in the United States and the United Kingdom. The item–total correlation of need for cognition ranged from .47 (Item 18) to .82 (Item 04) in the United States, and from .36 (Item 18) to .75 (Item 03) in the United Kingdom. The highest correlations were similar in both countries (Items 03 and 04).

**Table 1.** Descriptive Statistics, Item–Total Correlation, and Factorial Loadings.

	United States (α = .94)				United Kingdom (α = .91)			
Items	*M*	*SD*	*r*	Factor	*M*	*SD*	*r*	Factor
**NCS01**	**3.41**	**1.14**	.76**	**.75**	**3.28**	**1.11**	.70**	**.69**
**NCS02**	**3.57**	**1.15**	.78**	**.77**	**3.43**	**1.07**	.70**	**.71**
**NCS03** (R)	**3.94**	**1.10**	.81**	**.81**	**3.90**	**1.03**	.75**	**.75**
**NCS04** (R)	**3.65**	**1.14**	.82**	**.82**	**3.64**	**1.08**	.72**	**.71**
NCS05 (R)	3.83	1.13	.73**	.72	3.76	1.11	.64**	.60
NCS06	3.35	1.17	.73**	.70	3.09	1.13	.68**	.66
NCS07 (R)	3.58	1.21	.70**	.67	3.44	1.20	.64**	.60
NCS08 (R)	3.01	1.14	.61**	.57	2.97	1.03	.46**	.41
NCS09 (R)	3.06	1.22	.68**	.65	2.97	1.16	.62**	.57
NCS10	3.88	1.07	.72**	.71	3.53	1.08	.65**	.63
**NCS11**	**3.92**	**1.05**	.76**	**.75**	**3.79**	**.98**	.71**	**.70**
NCS12 (R)	3.99	1.09	.74**	.72	3.82	1.04	.62**	.59
NCS13	3.39	1.14	.68**	.65	3.20	1.16	.71**	.69
NCS14	3.78	1.11	.65**	.63	3.44	1.15	.60**	.57
**NCS15**	**3.62**	**1.11**	.76**	**.75**	**3.40**	**1.07**	.67**	**.65**
NCS16 (R)	3.20	1.26	.65**	.62	2.98	1.19	.52**	.47
NCS17 (R)	3.66	1.18	.65**	.61	3.39	1.16	.58**	.53
NCS18	3.69	1.13	.47**	.42	3.50	1.10	.36**	.29

*Note*. (R) = reversed items; *SD* = standard deviation. Items selected for the final version are given in boldface.

** *p* < .001.

##### Factorial structure

Initial screening allowed us to perform an EFA (United States: Kaiser–Meyer–Olkin [KMO] = .96, Bartlett’s test of sphericity = 8287.65(153), *p* < .001; United Kingdom: KMO = .95, Bartlett’s test of sphericity = 3413.60(153), *p* < .001). In both countries, one factor with eigenvalues greater than one emerged (Kaiser, 1960), indicating a unidimensional structure. To further strengthen the evidence for this structure, the original eigenvalues were compared with eigenvalues generated in simulated data (*k* = 1000), using the parallel analysis technique (Horn, 1965). In this technique, the structure is indicated by the number of eigenvalues that are higher in the actual data than in the simulated data. Once again, the results supported a one-factor structure in both countries (Çokluk & Koçak, 2016). The single factor explained 47.74% of the variance in the United States and 37.46% in the United Kingdom, in line with what was found in previous research (e.g., 37%, Cacioppo et al., 1984; 40%, Forsterlee & Ho, 1999; <29%, Georgiou & Kyza, 2019; 38.8%, Perri & Wolfgang, 1988). Next, we examined the factor loadings obtained through the EFA in both countries. As expected, most of the items presented good loadings (>.30), except Item 18 (.29) in the United Kingdom, which was marginally below this threshold (Table 1).

##### Discrimination and thresholds

We used IRT to compute discrimination and thresholds of the items. The parameter “discrimination” refers to the items’ ability to discriminate between individuals varying in the latent trait, helping to distinguish between those lower and those higher in need for cognition. Higher values indicate higher discrimination values. Table 2 presents the discrimination parameters for the full NCS. Following Baker’s (2001) discrimination classification, 14 items in the U.S. sample showed very high discrimination levels (*a* > 1.7), three items were high (1.35 < *a <* 1.69), and one was moderate (0.65 < *a <* 1.34). In the U.K. sample, nine items presented very high discrimination levels, five were high, three were moderate, and one was low (0.35 < *a <* 0.64).

**Table 2.** Item Parameters of the NCS in the United States and the United Kingdom.

	United Kingdom						United States					
Item	*a*	*b*1	*b*2	*b*3	*b*4	*b*(*m*)	*a*	*b*1	*b*2	*b*3	*b*4	*b*(*m*)
**NCS01**	**2.484**	**−1.885**	**−0.840**	**−0.164**	**1.197**	**−.423**	**2.145**	**−1.923**	**−0.800**	**−0.054**	**1.599**	**−.294**
**NCS02**	**2.588**	**−1.822**	**−0.967**	**−0.386**	**1.004**	**−.543**	**2.140**	**−2.213**	**−0.950**	**−0.197**	**1.395**	**−.491**
**NCS03**	**3.198**	**−2.153**	**−1.180**	**−0.765**	**0.394**	**−.926**	**2.420**	**−2.572**	**−1.385**	**−0.766**	**0.579**	**−1.036**
**NCS04**	**3.105**	**−2.037**	**−0.940**	**−0.412**	**0.756**	**−.658**	**2.116**	**−2.371**	**−1.172**	**−0.451**	**0.993**	**−.750**
NCS05	2.313	−2.334	−1.148	−0.683	0.565	−.900	1.648	−2.678	−1.395	−0.720	0.814	−.995
NCS06	2.032	−1.815	−0.821	−0.137	1.339	−.359	1.736	−1.837	−0.642	0.244	2.001	−.059
NCS07	1.899	−2.284	−0.944	−0.373	0.800	−.700	1.459	−2.420	−0.970	−0.332	1.284	−.609
NCS08	1.401	−2.126	−0.502	0.405	2.175	−.012	0.884	−3.426	−0.733	0.845	3.608	.074
NCS09	1.796	−1.846	−0.365	0.226	1.536	−.112	1.435	−2.258	−0.322	0.422	1.926	−.058
NCS10	2.229	−2.248	−1.381	−0.783	0.635	−.944	1.828	−2.187	−1.183	−0.398	1.342	−.607
**NCS11**	**2.400**	**−2.274**	**−1.385**	**−0.839**	**0.601**	**−.974**	**2.131**	**−2.604**	**−1.479**	**−0.749**	**0.963**	**−.967**
NCS12	2.358	−2.393	−1.381	−0.905	0.343	−1.084	1.546	−3.183	−1.551	−0.794	0.862	−1.167
NCS13	1.714	−2.089	−0.975	−0.142	1.472	−.434	2.043	−1.801	−0.660	0.085	1.561	−.204
NCS14	1.755	−2.336	−1.463	−0.774	0.833	−.935	1.409	−2.432	−1.157	−0.259	1.441	−.602
**NCS15**	**2.459**	**−2.132**	**−1.065**	**−0.351**	**0.874**	**−.669**	**1.913**	**−2.470**	**−0.976**	**−0.071**	**1.355**	**−.541**
NCS16	1.581	−1.820	−0.649	0.046	1.412	−.253	1.065	−2.208	−0.639	0.448	2.535	.034
NCS17	1.569	−2.671	−1.116	−0.533	0.852	−.867	1.161	−2.968	−1.015	−0.185	1.657	−.628
NCS18	0.919	−3.527	−1.862	−0.986	1.450	−1.231	0.590	−5.385	−2.166	−0.887	3.063	−1.344

*Note*. NCS = Need for Cognition Scale. *a* = discrimination; *b*1-*b*4 = threshold; *b*(*m*) = means across *b*1-*b*4. Items selected for the final version are given in boldface.

Next, the difficulty level of the items was assessed using an item threshold analysis. This analysis indicates the level of the latent trait that the individual needs to endorse to select the next higher option category. More difficult items tend to be endorsed only by individuals that present higher levels in the latent trait, whereas easier items tend to be endorsed by a wider range of individuals. Items should neither be too easy (e.g., means across b1-b4 < **−**1.5) nor too difficult (e.g., means across b1-b4 > **−**1.5) to be endorsed by the individual, as indicated by means across difficulty levels (Rauthmann, 2013). The difficulty levels of the items and the b1-to-b4 means can be seen in Table 2.

##### Item information curves

**Figure 1.**
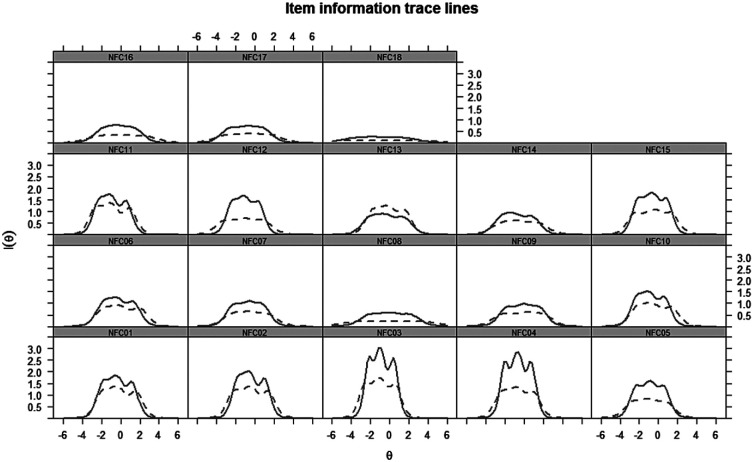
Item information curves, for USA and UK (dashed lines).

Item information curves (IICs) test how much information an item shares with the total information of the measure (Castro, Trentini, & Riboldi, 2010). Items with a higher *I*(θ), and thus, a higher curve are more informative whereas items with a flat curve carry only little information. Assessing the IIC is important because higher informativeness of items indicates higher measurement precision, less measurement error, and thus, higher reliability of the scale (Rauthmann, 2013). The IICs can be seen in Figure 1.

#### Developing the NCS-6

To create a shorter version of the NCS, we considered items that consistently performed well across the United States and the United Kingdom in CTT and IRT. Specifically, we retained items that contributed more to the full scale (i.e., *r_it_ >* .40*)*. Furthermore, we only retained items with factorial loadings above the recommended threshold of .30. Moreover, we used the same IRT-based criteria as previous research to determine which items to retain (e.g., Coelho et al., 2018; Rauthmann, 2013). The assessment of the item-discrimination levels revealed that nine items (Items 01, 02, 03, 04, 06, 10, 11, 13, 15) from the NCS were highly discriminative (*a* > 1.7) in both samples. The difficulty levels of these items were also in the recommended range (i.e., between ±1.5 and 0, Rauthmann, 2013), and thus, neither too easy nor too difficult. Finally, we examined the amount of information these nine items were contributing to need for cognition (Figure 1), using the IIC. We excluded the less informative items with *I*(θ) < 1 in both countries, leaving six items. Thus, we selected these six items (Items 01, 02, 03, 04, 11, and 15) to construct the very short scale. Given their better discriminative ability and informativeness, these six items should therefore be more reliable than the remaining 12 items; we tested this assumption below.

To further validate the six selected items, we calculated new discrimination and threshold parameters for these items (Table 3). All items were highly discriminative^2^ and presented good difficulty levels.

**Table 3.** Item Parameters of the NCS-6 in the United States and the United Kingdom.

	United States				United Kingdom							
Item	*a*	*b*1	*b*2	*b*3	*b*4	*b*(*m*)	*a*	*b*1	*b*2	*b*3	*b*4	*b*(*m*)
NCS01	2.523	−1.887	−0.850	−0.168	1.200	−.426	2.196	−1.900	−0.791	−0.049	1.599	−.285
NCS02	2.711	−1.804	−0.967	−0.387	1.002	−.539	2.618	−2.061	−0.892	−0.190	1.299	−.461
NCS03	2.712	−2.253	−1.243	−0.811	0.419	−.972	2.034	−2.778	−1.469	−0.814	0.634	−1.107
NCS04	2.792	−2.107	−0.987	−0.433	0.792	−.684	1.804	−2.570	−1.268	−0.482	1.075	−.811
NCS11	2.418	−2.274	−1.396	−0.855	0.604	−.980	2.229	−2.571	−1.456	−0.735	0.954	−.952
NCS15	2.728	−2.074	−1.048	−0.346	0.860	−.652	2.004	−2.427	−0.963	−0.063	1.341	−.528

*Note*. NCS = Need for Cognition Scale; *a* = discrimination; *b*1-*b*4 = threshold; *b*(*m*) = means across *b*1-*b*4.

**Figure 2.**
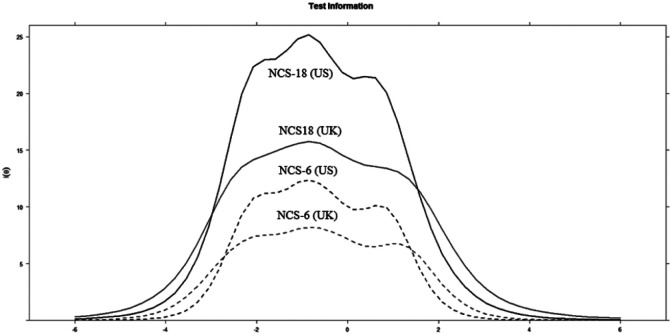
Test Information Curves for US and UK. *Note*. Solid line, NCS-18; dashed line, NCS-6. NCS = Need for Cognition Scale.

Finally, test information curves were assessed for the full and the short version of the NCS, separately for both countries (Figure 2). The test information curve is based on the amount of information all items add to the total, so it is expected that a shorter version is less informative. The curve is directly related to the reliability of the measure, with an information of 10 being equivalent to a reliability of .90 (Cappelleri et al., 2014). Overall, the results suggest a reasonable spread of discrimination across the latent range of need for cognition. The NCS-18 and NCS-6 correlated highly in the U.K. sample, *r*(474) = .93, *p* < .001, and in the U.S. sample, *r*(819) = .96, *p* < .001. However, because the NCS-6 items are a subset of the NCS-18 items, these correlations are likely to be inflated. We therefore correct for this (redundant) error variance, using Levy’s (1967) correction. The corrected coefficients were *r* = .77 and *r* = .85.

##### Confirmatory factor analysis

We performed a CFA in each country to test whether the six items of the NCS-6 also support the proposed one-factor structure (Cacioppo & Petty, 1982). While individual cutoff values may be biased, the NCS-6 consistently revealed a good model fit across all indices, providing strong support for the one-factor structure. For comparisons, we also added the model fit indices for the NCS-18, which showed slightly worse results, as can be seen in Table 4. The AIC and BIC revealed a better fit for the NCS-6 than the NCS-18.

**Table 4.** Model Fit Indices for the NCS-18 and NCS-6 in the United States and the United Kingdom.

		χ^2^(gl)	CFI	TLI	RMSEA [90% CI]	AIC	BIC
United States	NCS-18	628.15 (*p* < .001)	.92	.91	.067 [.062, .071]	38355	38610
	NCS-6	55.96 (*p* < .001)	.97	.95	.080 [.063, .097]	12454	12539
United Kingdom	NCS-18	410.49 (*p* < .001)	.89	.88	.065 [.059, .072]	23084	23309
	NCS-6	20.65 (*p* < .05)	.98	.97	.052 [.028, .077]	7388	7463

*Note*. CI = confidence interval; NCS = Need For Cognition Scale; CFI = comparative fit index; TLI = Tucker–Lewis index; RMSEA = root mean square error of approximation; AIC = Akaike information criterion; BIC = Bayesian information criterion.

##### Reliability

The reliabilities in both countries were good (United States: McDonald’s ω and Cronbach’s α = .90; United Kingdom: ω and α = .86; Kline, 2013) and comparable with the NCS-18 (United States: McDonald’s ω and Cronbach’s α = .94; United Kingdom: ω and α = .91). Furthermore, the reliabilities of the NCS-6 were also comparable with the reliabilities of the excluded 12 items (United States: ω and α = .89; United Kingdom: ω = .85 and α = .84). Because the reliability indices often increase with a larger number of items, we additionally computed the average correlations between the 6 selected items and between the 12 excluded items, respectively. As expected, the interitem correlations were on average larger for the selected 6 items than for the excluded 12 items (United States: *M_r_* = .60 vs. .41; United Kingdom: *M_r_* = .50 vs. .31).

##### Measurement invariance

We performed multigroup CFAs to assess if the NCS-6 is invariant across countries and gender. To do so, three models were considered (i.e., configural, metric, and scalar), with the results showing invariance across both country and gender in all models (Table 5). That is, the model fit did not decrease substantially when loadings and intercepts were forced to be invariant, suggesting that they are similar across countries and gender.

**Table 5.** Measurement Invariance of the NCS-6 Across Countries and Gender.

	Models of invariance	CFI	RMSEA	ΔCFI	ΔRMSEA
Country	Configural	.975	.070	—	—
United States (*n* = 821); United Kingdom (*n* = 476)	Metric	.972	.065	.002	.005
	Scalar	.968	.064	.005	.001
Gender	Configural	.972	.073	—	—
Female (*n* = 606); male (*n* = 672)	Metric	.970	.068	.002	.006
	Scalar	.961	.069	.008	.002

*Note*. NCS = Need for Cognition Scale; CFI = comparative fit index; RMSEA = root mean square error of approximation. Δ = differences between the current and the previous model.

#### Construct Validity of the NCS-6

##### Convergent validity

To assess the convergent validity, we correlated both the full and the short version of the NCS with several constructs to test whether the correlations of the NCS-18 and NCS-6 with other constructs are similar. The correlational analyses supported this (Table 6): Both the NCS-18 and NCS-6 correlated in very similar ways with a range of important psychological variables. As expected, the NCS-6 was positively correlated with need for affect, self-direction values, social desirability, and attitudes toward stereotypically cold and competent groups and toward competence attributes. In contrast, the NCS-6 was negatively correlated with conformity values.

**Table 6.** Correlations With Convergent Constructs, Using the NCS-18, NCS-12 (12 Excluded Items), and NCS-6.

Country	*N*	Construct	NCS-18	NCS-12	NCS-6
United States (total *n* = 821)	821	Approach (NFA)	.17**	.17**	.16**
	821	Avoidance (NFA)	−.21**	−.23**	−.17**
	821	Overall (NFA)	23**	.25**	.20**
	221	Social Desirability	.23**	.23**	.22**
	331	Competence	.13*	.13*	.14*
	331	Warmth	.05	.06	.03
	440	Attitudes cold and competent groups	.15**	.15**	.14**
	440	Attitudes warm and incompetent groups	.03	.03	.02
United Kingdom (total *n* = 476)	476	Approach (NFA)	.21**	.19**	.23**
	—	Avoidance (NFA)	−.27**	−.26**	−.26**
	—	Overall (NFA)	.30**	.28**	.30**
	—	Security	−.20**	−.20**	−.17**
	—	Conformity	−.23**	−.23**	−.17**
	—	Benevolence	.17**	.15**	.19**
	—	Universalism	.36**	.36**	.32**
	—	Self-direction	.42**	.40**	.41**
	—	Stimulation	.31**	.27**	.33**
	—	Hedonism	.08	.06	.11*
	—	Achievement	.19**	.16**	.22**
	—	Power	−.06	−.08	−.00

*Note*. NCS = Need for Cognition Scale; NFA = Need for Affect.

* *p <* .05. **p < .01.

##### Discriminant validity

As expected, the NCS-6 was unrelated to attitudes toward stereotypically warm and incompetent groups and toward warmth attributes.

##### Summary

Overall, the difference in magnitude of correlations between the NCS-18 and NCS-6 was ⩽.01 for 7 out of the 20 comparisons across both samples (Table 6), between .01 and .05 for 11 further comparisons, and only for 2 comparisons .06 (conformity and power values). Thus, the cost of using the NCS-6 in terms of decreased validity is generally very small. Across both samples, 17 of the correlations between the NCS-18 and NCS-6 and other psychological variables were statistically significant. In 6 out of the 17 cases, the correlations of the NCS-6 were slightly stronger, in 10 cases slightly weaker. The correlation of the NCS-6 with the value-type hedonism reached statistical significance, whereas the correlation of the NCS-18 with hedonism did not (*r =* .11 vs. .08). The correlations of the 12 excluded items were similar to the correlations of the two other scales (Table 6). While this finding indicates that even the excluded items show good construct validity, the NCS-6 achieved a similar level of construct validity with fewer items and superior interitem correlations.

## Study 2

Despite the informative character of the EFA in Study 1, performing both EFA and CFA in the same samples can lead to overfitting of the results (Fokkema & Greiff, 2017). Thus, Study 2 aimed to confirm the structure of the NCS-6 in an independent sample, to further demonstrate the convergent validity of the NCS-6, and to compare the NCS-6 with a previously developed unvalidated two-item version of the NCS (NCS-2). To examine the convergent validity of the NCS-6, we included a range of variables that were previously found to be associated with need for cognition: the Big-5 personality trait openness to new experience (e.g., Tuten & Bosnjak, 2001), cognitive reflection test (Frederick, 2005; Pennycook, Cheyne, Koehler, & Fugelsang, 2016; Toplak, West, & Stanovich, 2014), interests in politics (Bizer et al., 2000; Cacioppo, Petty, Kao, & Rodriguez, 1986), education (Cacioppo & Petty, 1982), and visiting museums (Packer & Ballantyne, 2002). To test for discriminant validity, we included political ideology, which was previously found to be unrelated to need to cognition (Federico & Schneider, 2007). Finally, we were also interested in whether using the NCS-6 saves a significant amount of time compared with the NCS-18.

### Method

#### Participants

We aimed to broadly match the sample to the British sample of Study 1 in terms of representativeness and country of origin to be able to compare the completion time of the NCS-18 with the NCS-6 and NCS-2. Participants were 299 individuals (219 women, 78 men, 2 other; *M*_age_ = 37.55 years, *SD* = 11.78) who were living in the United Kingdom and were recruited online through Prolific academic (prolific.ac) from the general population. One participant reported that primary school was the highest completed educational level, 9 secondary school, 38 GCSE or similar, 90 A-level or similar, 108 undergraduate education, and 53 completed a postgraduate education. The study was approved by the ethics committee and collected together with an unrelated study (examining attitudes toward children).

#### Material

##### Need for Cognition

We measured need for cognition with the NCS-6 (see the appendix for the items). Additionally, we included a two-item scale of need for cognition (NCS-2) which is used in the American National Election Survey but has not been formally validated, except that its construct validity was demonstrated (Bizer et al., 2000). The two items of the NCS-2 were chosen based on the highest loading items of Cacioppo and Petty’s (1982) original factor analysis and read

Some people like to have responsibility for handling situations that require a lot of thinking, and other people don’t like to have responsibility for situations like that. What about you? Do you like having responsibility for handling situations that require a lot of thinking, do you dislike it, or do you neither like nor dislike it?

and

Some people prefer to solve simple problems instead of complex ones, whereas other people prefer to solve more complex problems. Which type of problem do you prefer to solve: simple or complex?

We aimed to compare the NCS-6 with the NCS-2 in terms of reliability and construct validity.

##### Openness to new experiences or intellect (Goldberg, 1992)

This construct was measured with a seven-item bipolar scale. Participants were asked to describe how they see themselves at the present time on a scale ranging from 1 (e.g., *very unreflective*) to 9 (e.g., *very reflective*; α = .74).

##### Cognitive reflection test (Frederick, 2005)

This test measures cognitive ability with three items that have an intuitive but wrong answer and a correct answer, including “If it takes 5 machines 5 minutes to make 5 widgets, how long would it take 100 machines to make 100 widgets?”

##### Interest in politics

Interest in politics was measured with three items we created for this study: “How interested are you in British politics?” “How closely are you following the recent political developments?” and “How closely are you following the news?” (α = .91). Answers were given on a 7-point scale ranging from 1 (*not at all*) to 7 (*very much*).

##### Education

Participants responded to the item “What is the highest level of education you have completed?” on a 7-point scale ranging from 1 (*no schooling completed*) to 7 (*postgraduate education*).

##### Interest in museums

Participants interest in museums was measured with two items we created for this study: *How often do you visit museums?* and *How much do you enjoy visiting museums?* Responses were given on a 5-point scale ranging from 1 (*never/ not at all*) to 5 (*all the time/ very much*). Both items correlated with *r*(297) = .40, *p* < .001 (α = .47) and were averaged.

##### Political orientation

Political orientation was measured with a 11-point scale ranging from 0 (*left*) to 10 (*right*) with 5 (*center*) being the scale midpoint. The distribution of the responses was approximately normal, with the mode being 5.

### Results

#### Confirmatory Factor Analysis

We performed a CFA to test whether the unidimensional structure of the NCS-6 replicated. The model fit was similar to the one found in Study 1, χ^2^(9) = 24.88 (*p* = .003), CFI = .97, TLI = .95, RMSEA =.077 (95% confidence interval [CI] = [.049, .106]), AIC = 4773, BIC = 4839, with lambdas varying between .82 (Item 2) and −.70 (Item 3). The reliabilities were good (ω and α = .87; Kline, 2013). The reliability of the NCS-2 was good as well: Both items correlated with *r*(297) = .51, *p* < .001 (ω = .74 and α = .58). The NCS-6 and NCS-2 correlated with *r*(297) = .83, *p* < .001.

#### Convergent Validity

Table 7 displays the correlations between the NCS-6 and NCS-2 with all five variables. The correlations of the NCS-6 with all variables were slightly, but consistently higher than the correlations of the NCS-2. Also, the strength of the correlations was very similar to previous research that relied on the NCS-18. For example, we found a correlation of .45 between openness and the NCS-6, compared with Tuten and Bosnjak’s (2001) correlation of .37 using the NCS-18. Furthermore, we found a correlation of .26 between the cognitive reflection test and the NCS-6, whereas previous research reported correlations between these constructs ranging from .22 to .28 (Frederick, 2005; Pennycook et al., 2016; Toplak et al., 2014).

**Table 7.** Correlations With Convergent Constructs, Using the NCS-6 and NCS-2.

Construct	NCS-6	NCS-2
Openness/intellect	.45***	.36***
Cognitive reflection test	.26***	.21***
Interest in politics	.27***	.25***
Education	.30***	.26***
Interest in museums	.25***	.19**
Political orientation	−.05	−.05

*Note. N* = 299 (for political orientation: *N* = 288). NCS = Need for Cognition Scale.

* *p <* .05. **p < .01. ***p < .001.

#### Discriminant Validity

As expected, the NCS-6 and the NCS-2 were both unrelated to political orientations (Table 7).

#### Time Required for Scale Completion

Finally, we tested whether the NCS-6 saves participation time compared with the NCS-18. In Study 1, we timed how long it took for participants of the British sample to complete the NCS-18 (in seconds). The sample of Study 2 was drawn from the same participant pool, making comparisons meaningful. Additionally, we also timed (in seconds) how long it took participants to complete the NCS-2. Participant took longest to complete the NCS-18 (*M* = 114.52, *SD* = 94.09, *Mdn* = 89.93), were faster in completing the NCS-6 (*M* = 43.42, *SD* = 35.49, *Mdn* = 36.03), and fastest in completing the NCS-2 (*M* = 21.15, *SD* = 20.02, *Mdn* = 17.24). The average completion time per item was 6.36, 7.24, and 10.58 seconds, respectively. If researchers wish to pay their participants US$10 (which is roughly the minimum wage in several Western countries in 2018), the NCS-6 saves US$0.20 and the NCS-2 saves US$0.26 compared with the NCS-18 per participant. These estimates are excluding service fees charged by survey websites such as MTurk and Prolific, which currently range between 30% and 40%. This estimate of the time saved is likely to be conservative because samples recruited through survey websites such as MTurkor prolific have more experiences in completing online surveys and are therefore faster than people with less or no experience.

## General Discussion

Long measures in a survey can be problematic given that they may increase participant fatigue, lack of attention, boredom, and dropouts (e.g., Rammstedt & Beierlein, 2014), which has ethical implications and can compromise the results. This may be particularly the case for relatively complicated measures as the NCS, and accordingly, researchers have resorted to using unvalidated shortened versions of the NCS (e.g., Bizer et al., 2000; Bullock, 2011; Davis et al., 1993). Thus, given the importance of need for cognition in the literature, we propose a carefully validated shorter version based on data from two countries.

We developed the very short NCS-6 using a comprehensive approach that combines IRT and classic test theory. Across three large samples, the NCS-6 showed excellent psychometric properties, including strong evidence of its convergent and discriminant validity. In particular, the NCS-6 is highly correlated with the NCS-18, and the pattern of correlations with external psychological variables were similar for both scales and in line with previous research using the NCS-18. For example, the NCS-6 correlated .45 with openness (cf. *r* = .37 in Tuten & Bosnjak, 2001) and .26 with the cognitive reflection test (cf. .22-.28 in Frederick, 2005; Pennycook et al., 2016; Toplak et al., 2014). Moreover, the findings indicate that the cost in construct validity by using the NCS-6 rather than the NCS-18 is generally very small: In 6 out of the 17 significant correlations in Study 1, the correlations of the NCS-6 were slightly stronger, in 10 cases slightly weaker.

Corroborating these findings, Edwards (2009) assessed the quality of the NCS-18 items using IRT and also found that the six items selected by us had the highest discrimination levels and recommended difficulty levels. Edwards’s analysis served as an example to demonstrate IRT but was never used to propose a shortened NCS and hence lacked important tests of reliability and validity. Nevertheless, this past evidence, which is based on a sample of 3,364 individuals drawn from 30 studies, provides further support for the robustness of the NCS-6 items.

Concerning time savings, participants were on average 70 seconds, or almost three times, faster in completing the NCS-6 than the NCS-18, thus saving valuable time and potentially reducing participant fatigue and enhancing the data quality particularly for longer surveys. These time savings satisfy an apparent need for a very short measure of the need for cognition, as evidenced by the use of unvalidated shortened scales in the literature. In addition to being unvalidated, some of these scales have shown methodological shortcomings (Ebersole et al., 2016; Luttrell et al., 2017) and failed to reproduce classic effects (Bakker & Lelkes, 2016; Bizer et al., 2000). Moreover, in the present research, the NCS-6 slightly but consistently outperformed the NCS-2 in terms of its convergent validity. Hence, overall, the present findings demonstrate that the NCS-6 is a reliable and valid scale, making it a useful and widely applicable measure of the need for cognition. To save time and money, the NCS-6 can be administered in place of the NCS-18 with only very minor costs to reliability and validity.

Furthermore, we gathered evidence for the scale’s measurement invariance. Previous research has shown that the NCS-18 is gender-neutral and shows similar factorial structures in both Europe and North America (Cacioppo et al., 1996). Thus, demonstrating that our shortened NCS similarly allows for meaningful comparisons across these groups, we found evidence that the NCS-6 is invariant across gender and across the United Kingdom and the United States. Although the two samples in Study 1 were largely comparable, it is noteworthy that they also differed in terms of recruitment method (i.e., Prolific, MTurk), age (i.e., the U.K. sample was somewhat older), gender distribution (i.e., fewer men in the U.K. sample), and additional measures that were assessed in the surveys. However, differences between recruitment methods are unlikely to have influenced the findings: Previous research found that several effects were consistently replicated across both recruitment methods (i.e., Prolific and MTurk; Peer, Brandimarte, Samat, & Acquisti, 2017). Hence, while it is important to keep in mind that our test for invariance across samples did not exclusively compare nationality, obtaining support for the NCS being invariant across these samples despite these additional differences further attests to the reliability of the NCS-6 in different contexts. Nevertheless, it would be useful to conduct further research on the measurement invariance of NCSs across other relevant individual difference variables such as education, political and religious orientations, and income.

Future research may benefit from testing the scale’s applicability in other countries. That is, given that our data were derived from two Western countries, the United States and the United Kingdom, we cannot make claims about the measure’s applicability in non-Western countries. In fact, previous validation studies in other countries or languages have excluded one or several items from the overall NCS based on low factor loadings, even in other Western countries such as Australia (Forsterlee & Ho, 1999), Germany (Bless et al., 1994), but also in Greece (Georgiou & Kyza, 2019) or in a U.S.-American sample of Hispanics (Culhane et al., 2004). While it is noteworthy that none of the items maintained in the NCS-6 were dropped in other cultures, these findings suggest that the full NCS may not be invariant across countries other than the United States and the United Kingdom. Thus, it may be fruitful for future research to test whether the NCS-6 is a reliable and valid measure in various countries, to test how widely applicable the NCS-6 is.

Finally, our samples were, although not representative, drawn from the general public. Previous research has found that samples from the two survey platforms we used, MTurk and Prolific Academic, are similar to the results obtained in student samples and in nationally representative population-based samples (Mullinix, Leeper, Druckman, & Freese, 2015; Paolacci, Chandler, & Ipeirotis, 2010). While this suggests that our findings are to some extent generalizable to the general public, future research would benefit from examining the generalizability in more detail using representative samples.

The present research provides an important contribution by introducing a very short scale to measure the need for cognition (NCS-6). We found strong psychometric evidence for the use of the NCS-6 across the United States and the United Kingdom. Together with established measurement invariance and meaningful correlations with other psychological constructs, our findings indicate that the NCS-6 is a parsimonious, reliable, and valid measure of need for cognition which may benefit future research.

## Appendix

### Items That Compose the Need for Cognition Scale–6 (NCS-6)

01. I would prefer complex to simple problems.
02. I like to have the responsibility of handling a situation that requires a lot of thinking.
03. Thinking is not my idea of fun. (R)
04. I would rather do something that requires little thought than something that is sure to challenge my thinking abilities. (R)
11. I really enjoy a task that involves coming up with new solutions to problems.
15. I would prefer a task that is intellectual, difficult, and important to one that is somewhat important but does not require much thought.

## Notes

1. The use of cutoff values for goodness-of-fit indices has been questioned, because the indices are dependent on a range of “nuisance” factors, including sample size and number of items (e.g., F. Chen, Curran, Bollen, Kirby, & Paxton, 2008; Greiff & Heene, 2017). Thus, although larger sample sizes (>200 participants) provide more accurate estimates of fit indices, specifying thresholds remains problematic (F. Chen et al., 2008). To avoid relying on individual cutoff values which may be biased, we followed F. Chen et al.’s recommendations to use multiple fit indices alongside “human judgement.”

2. Interestingly, in the context of the NCS-18, the NCS-6 items showed higher overall discrimination levels in the U.S. sample (i.e., *a*s = 2.4-3.198) than the NCS-6 items in the U.K. sample (i.e., *a*s = 1.913-2.42). This indicates that the NCS-6 items in the context of all NCS-18 items had a higher ability to distinguish individuals in the U.S. sample than in the U.K. sample. Importantly, however, when the NCS-6 items were analyzed in isolation, the U.S. sample showed a narrower range of discrimination levels (i.e., *a*s = 2.418-2.792) and was more comparable to the U.K. sample (i.e., *a*s = 1.804-2.618). Hence, the NCS-6 items in isolation appear to be more similar to each other in terms of their discrimination ability than the same items when considered in comparison with the full 18-item scale. The items’ higher consistency in discriminating across groups potentially points to another advantage of shortening the NCS.
